# Structure-Immunogenicity Relationship of α- and β-Tetrasaccharide Glycoforms from *Bacillus anthracis* Exosporium and Fragments Thereof

**DOI:** 10.3390/molecules23082079

**Published:** 2018-08-20

**Authors:** Riccardo De Ricco, Christy L. Ventura, Filippo Carboni, Rina Saksena, Pavol Kováč, Roberto Adamo

**Affiliations:** 1 GSK, Research Centre, via Fiorentina 1, 53100 Siena, Italy; riccardo.x.de-ricco@gsk.com (R.D.R.); filippo.x.carboni@gsk.com (F.C.); 2 Department of Microbiology and Immunology, Uniformed Services University of the Health Sciences, Bethesda, MD 20814, USA; christy.ventura.ctr@usuhs.edu; 3 NIDDK, LBC, National Institutes of Health, Bethesda, MD 20892-0815, USA; rsaksena@unm.edu (R.S.); kovac@niddk.nih.gov (P.K.)

**Keywords:** carbohydrates, glycoconjugates, *B. anthracis*, anthrose, BclA, vaccines, diagnostics

## Abstract

The tetrasaccharide (2-*O*-methyl-4-(3-hydroxy-3-methylbutamido)-4,6-dideoxy-α-d-glucopyranosyl-(1→3)-α-l-rhamnopyranosyl-(1→3)-α-l-rhamnopyranosyl-(1→2)-l-rhamnopyranose) from the major exosporium protein (BclA) of *Bacillus anthracis* has been proposed as a target for development of diagnostics and immune therapy or prophylaxis. While the immunodominant character of the anthrose residue has been previously elucidated, the role of the stereochemical configuration of the downstream rhamnose is unknown. Because the linkage of this residue to the GlcNAc bridging the glycan and the protein is lost during isolation of the tetrasaccharide, its α- and β-glycoforms have been synthesized. Herein, we prepared neoglycoconjugates from a series of fragments of the tetrasaccharide, including the complete α- and β-tetrasaccharide glycoforms, a 2-demethoxylated version of the α-tetrasaccharide, and the α- and β-trirhamnosides and CRM_197_. By immunization of mice, we showed that the anti α- and β-tetrasaccharide serum equally recognized both glycoforms. In contrast the sera produced following immunization with the α- and β-trirhamnoside fragments exhibited higher recognition for their own antigens than for their anomeric counterparts. The anti α- and β-tetrasaccharide sera recognized Sterne spores in a comparable fashion. ΔBclA spores not expressing the major exosporium protein were also recognized by the same sera, while mutants that produced the carbohydrate antigen with deletion of either rhamnose or anthrose were not. The tetrasaccharide could, therefore, be expressed in proteins other than BlcA. This work proves that α- and β-tetrasaccharide are equally potent immunogens.

## 1. Introduction

*Bacillus anthracis*, the causative agent of anthrax in humans and other mammals, is a Gram-positive, rod-shaped spore forming bacterium [1]. Due to the low number of spores (2500–55,000) that constitute the proposed median lethal inhalation dose (LD_50_) for humans [2], the potential of this bacterium as a bioterrorism weapon is dramatically high [3]. The main virulence factor produced by *B. anthracis* is the anthrax toxin, a three-protein exotoxin composed of the edema factor (EF), the lethal factor (LF), and the protective antigen (PA). These three proteins are individually nontoxic, but when acting in binary combinations, they can induce edema and cell death in the host [4]. Currently available vaccines based on attenuated nonencapsulated toxin-producing *B. anthracis* as well as PA-based vaccines are limited in their immunogenicity and efficacy; therefore, new antigens and novel formulations are needed.

If inhaled, anthrax spores can germinate in the lymphatic system and further spread via the bloodstream. After initial nonspecific symptoms, such as fever, malaise, and fatigue, severe respiratory distress with dyspnea, stridor, and cyanosis appear within 2 to 3 days, leading to respiratory failure due to bacteremia often associated with meningitis and subarachnoid hemorrhage [5]. Hence, targeting the spores could be crucial to combat the rapid bacterial replication.

The major surface component of the exosporium, which is the primary permeability barrier of the spore, is the highly immunogenic glycoprotein BclA, which has a long, central collagen-like region with multiple XXG repeats, most of which are PTG triplets [5]. BclA is known to be O-glycosylated in the threonine residue of these triplet repeats by a 715-Da tetrasaccharide, which is likely attached to the protein through a GalNAc residue [6]. 

**Figure 1:**
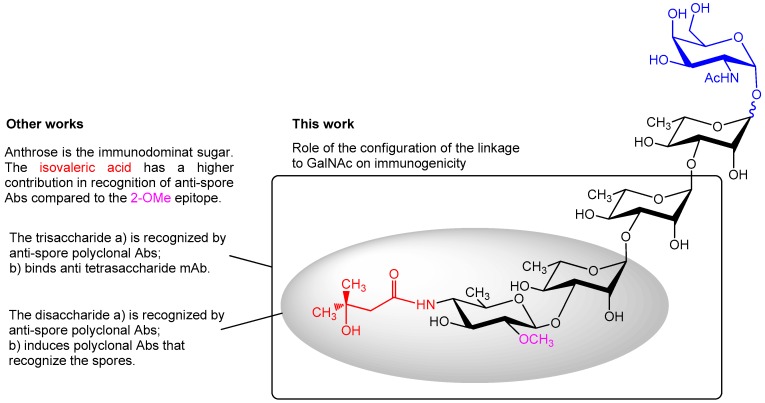
Chemical structure of the glycan decorating the exosporium BclA protein of *B. anthracis* and summary of the features contributing to the immunological properties of the tetrasaccharide portion. The square region denotes the trisaccharide portion of the glycan, while the grey oval area denotes the upstream disaccharide.

Compared to immunization with the carbohydrate antigens alone, immunization with neoglycoconjugates markedly enhances the specific anticarbohydrate immune responses. Therefore, conjugation of poorly immunogenic carbohydrates to carrier proteins is a well-established strategy for the prevention of many life threating diseases including meningitis and pneumonia, ensuring long lasting protection of infants, elderly, and immunocompromised populations [7,8]. Thus, a lot of effort has been put into using the BclA tetrasaccharide (2-*O*-methyl-4-(3-hydroxy-3-methylbutamido)-4,6-dideoxy-α-d-glucopyranosyl-(1→3)-α-l-rhamnopyranosyl-(1→3)-α-l-rhamnopyranosyl-(1→2)-l-rhamnopyranose, Figure 1) as a target for immune therapy or vaccination [9].

The 2-*O*-methyl-4-(3-hydroxy-3-methylbutamido)-4,6-dideoxy-d-glucose at the nonreducing terminal end of this oligosaccharide is known by the trivial name anthrose [6], and its structural identity was confirmed by chemical synthesis [10]. 

Due to its therapeutic potential, this bacterial glycan has inspired numerous syntheses which were recently reviewed [11,12]. Target of the first total synthesis of the conjugation-ready tetrasaccharide was a substance equipped with an α-linked spacer at the downstream end [13]. During the hydrazinolysis [6], which released the tetrasaccharide from the exosporium, the information regarding the stereochemistry of the linkage to GalNAc was lost. Therefore, the availability of both α and β glycoforms was deemed a prerequisite to understanding the relevance of this linkage in raising anti-exosporium antibodies. Accordingly, synthesis of not only α- but also of the β-linked tetrasaccharide was undertaken [14,15]. In order to identify the minimum structural requirements in the antigen that could be used as the antigenic component of a vaccine for anthrax, various fragments of the tetrasaccharides have also been synthesized, including oligosaccharides with the terminal β- or α-anthrose [16,17], trisaccharide analogues with variations at position 2 or 4, and a disaccharide containing the terminal non methylated analog of anthrose [18].

Other approaches towards the BclA tetrasaccharide focused on 1,2-*trans* installation of the terminal anthrose moiety through β-selective glycosylation [19], or on asymmetric synthesis from achiral compounds [20]. Substantial improvements in the synthesis of anthrose or related building blocks were achieved recently [21,22,23], and feasibility of grams-scale synthesis of the tetrasaccharide has been demonstrated [24]. The preparation of a pentasaccharide mimicking the tetrasaccharide linkage of to the GalNAc residue of BclA was also reported [25]. 

These synthetic studies [9,11,12] were undertaken with the aim to understand the potential role of the BclA glycan and related fragments as haptens for conjugation to proteins. The related in vitro studies aided the elucidation of the structural requirements for recognition of antis-pore antibodies. Conjugation of the synthetic tetrasaccharide to Keyhole limpet hemocyanin (KLH) was initially used for the generation of a carbohydrate specific mAb that recognized the glycan structure on the surface of *B. anthracis* [26]. An anthrose-containing trisaccharide conjugated to KLH [18] was shown to be sufficient for binding to anti-spore rabbit serum, and competitive ELISA with a trisaccharide lacking the C-4 isovaleric acid (3-hydroxy-3-methylbutanoyl) substituent highlighted the crucial role of this moiety in antibody recognition. In line with these results, glycoarray analysis demonstrated that—among a set of carbohydrates including anthrose, di-, and a tri-rhamnose backbone structures with and without anthrose, and the α- and β-tetrasaccharides—only structures that included the anthrose residue were recognized by the anti-spore rabbit sera [27]. 

Mapping studies on the anti-tetrasaccharide mAb involving combination of glycoarray, SPR and STD NMR experiments revealed that the side chain at position 4 of anthrose has a higher impact in antibody binding compared to the 2-*O*-methyl group [28]. 

Antibodies elicited against a truncated, demethylated anthrose-containing disaccharide conjugated to BSA [29] as well as an anthrose-KLH conjugate recognized intact *B. anthracis* spores [21]. While all these studies point out the immunodominant character of the anthrose residue and its structural requirements for antibody recognition, very little is known about how the configuration of the linkage to the GalNAc anchor contributes to the immunogenicity of the tetrasaccharide and the capacity of antibodies against the tetrasaccharide to recognize *B. anthracis* spores.

Here, the α- and β-tetrasaccharide glycoforms, a 2-demethylated version of α-tetrasaccharide, and the α- and β-trirhamnosides were conjugated to CRM_197_. Unlike other carrier proteins which require chemical detoxification, CRM_197_ is a 58 kDa mutant of diphtheria toxin, genetically detoxified by replacement of the glycine at position 52 with glutamic acid, and commonly used in the production of commercial vaccines [30]. 

The prepared conjugates served us in the exploration of the capacity of these oligosaccharides to generate, in mice, anti-spore antibodies. 

## 2. Results

### 2.1. Synthesis of the Glycoconjugates

**Figure 2:**
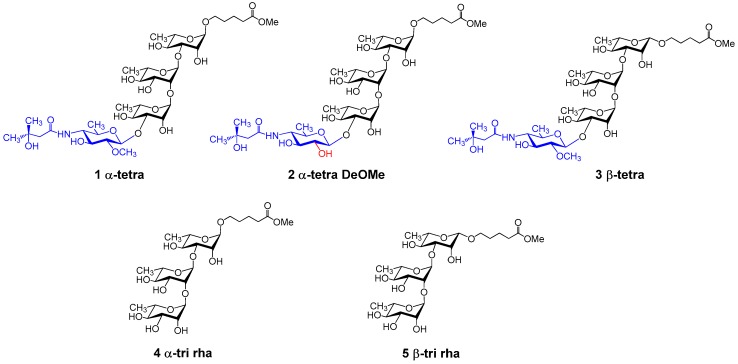
BclA glycan structures used in this study.

**Figure 3:**
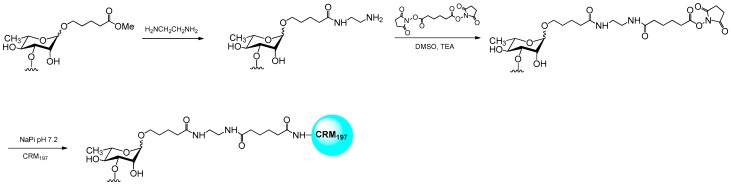
Oligosaccharide conjugation to CRM_197_.

To ascertain the differences in the immunogenicity of the α- and β-tetrasaccharide glycoforms, a series of compounds (Figure 2) were conjugated to CRM_197_. The glycans were synthesized with a 5-carboxylmethylpentyl linker as previously reported [9,15,16,17]. For conjugation, the fragments were first condensed with ethylenediamine, as described [17] (Figure 3). The amine was further extended by reaction with an excess of *N*-hydroxysuccinimidyl adipate in DMSO containing triethylamine, to obtain the active esters required for protein conjugation [31].

**Figure 4:**
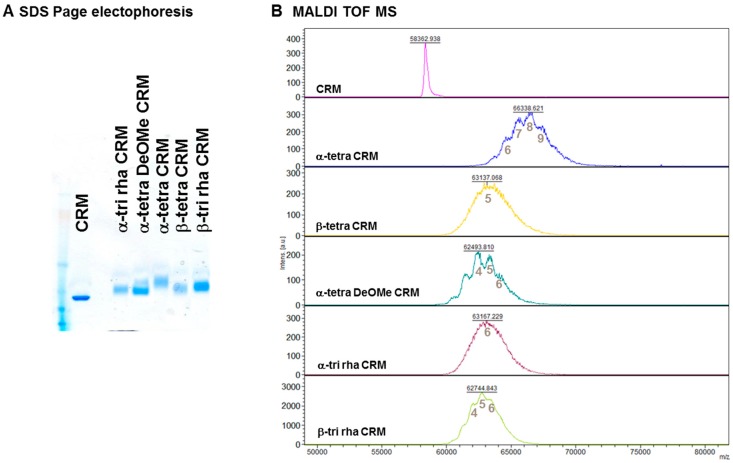
(**A**) SDS-PAGE (8–12%) and (**B**) MALDI-TOF MS spectra of synthesized glycoconjugates.

The intermediates were purified by precipitation with ethyl acetate and washed with the same solvent to remove the unchanged linker. After quantification of the active NHS groups, the purified oligosaccharide derivatives were incubated overnight with CRM_197_ using a 80–100:1 saccharide/protein molar ratio, corresponding to 40–50:1 NHS/protein molar ratio based on the spectrophotometric quantification of the active ester. Conjugation was verified by SDS-PAGE and MALDI-TOF MS analysis (Figure 4). All the conjugates showed a very homogeneous degree of sugar incorporation ranging from 5 to 7 carbohydrate moieties. Although the selected conjugation approach lead to moderate conjugation efficiency (10–17.5%) if compared to other reported methods [32], the advantage of the adipate linker consists in its immunosilent character.

### 2.2. Immunological Evalution

Groups of 8 BALB/c mice were immunized with the synthesized glycoconjugates by subcutaneous injection of a 2 µg dose (based on the saccharide) in the presence of Alum [Al(OH)_3_] as adjuvant. Control mice were immunized with PBS. One group was immunized with the conjugated α-tetrasaccharide in the absence of Alum to assess the effect of the adjuvant. Boosts were given at day 14 and 28 after the first injection. Sera were collected two weeks after the second boost and analyzed for anticarbohydrate antibodies against the α- and β-tetrasaccharide conjugated to BSA via squarate chemistry, in order to rule out possible detection of antispacer antibodies.

**Figure 5:**
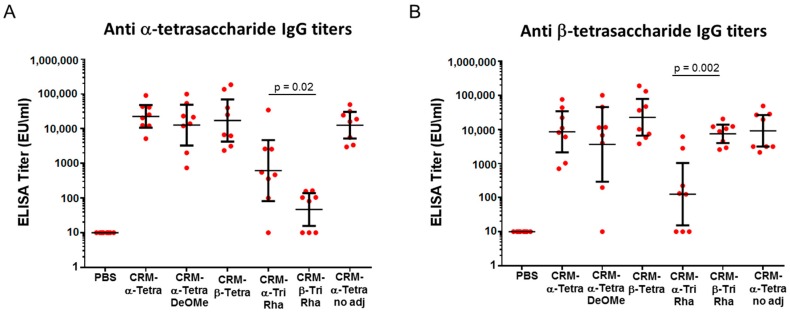
IgG levels after the second boost in mice immunized with the different conjugated fragments, with and without Alum, using α-tetrasaccharide-BSA (**A**) and β-tetrasaccharide-BSA (**B**) for coating. PBS was the negative control. Horizontal large bars indicate geometric mean titers (GMT) of each group, with 95% statistical confidence intervals indicated by small bars. Mann–Whitney analysis was used for statistics of α-Trirha-CRM_197_ vs. β-Trirha-CRM_197_.

The α- and β-tetrasaccharide induced comparable levels of IgGs against each other (Figure 5A). The removal of the 2-*O*-methyl group from α-tetrasaccharide did not impair the recognition compared to that of the methylated counterpart. In contrast, higher levels of anti-tetrasaccharide antibodies were observed in the serum from the α-trirhamnoside compared to the β-isomer (*p* = 0.02). These data indicate that when the anthrose residue is present, the immunogenicity is driven predominantly by this immunodominant sugar, while meaningful differences could be detected only with the backbone rhamnosides.

When the β-tetrasaccharide conjugated to BSA was used as coating reagent (Figure 5B), again no significant differences were found for the sera raised against the two tetrasaccharide glycoforms. Conversely, the β-trirhamnoside gave higher IgG titers compared to the α-isomer (*p* = 0.002). 

In both cases, administration of the α-tetrasaccharide conjugate without adjuvant did not diminish the level of anti-tetrasaccharide antibodies, indicating that the conjugated glycan is strongly immunogenic even in absence of the adjuvant.

Overall, these results indicate that the immune response elicited by the conjugated tetrasaccharide is primarily influenced by anthrose and is independent of the presence of the 2-*O*-methyl substituent. However, specificity for the terminal anomer can be detected with the trirhamnose backbone portions lacking the anthrose residue.

**Figure 6:**
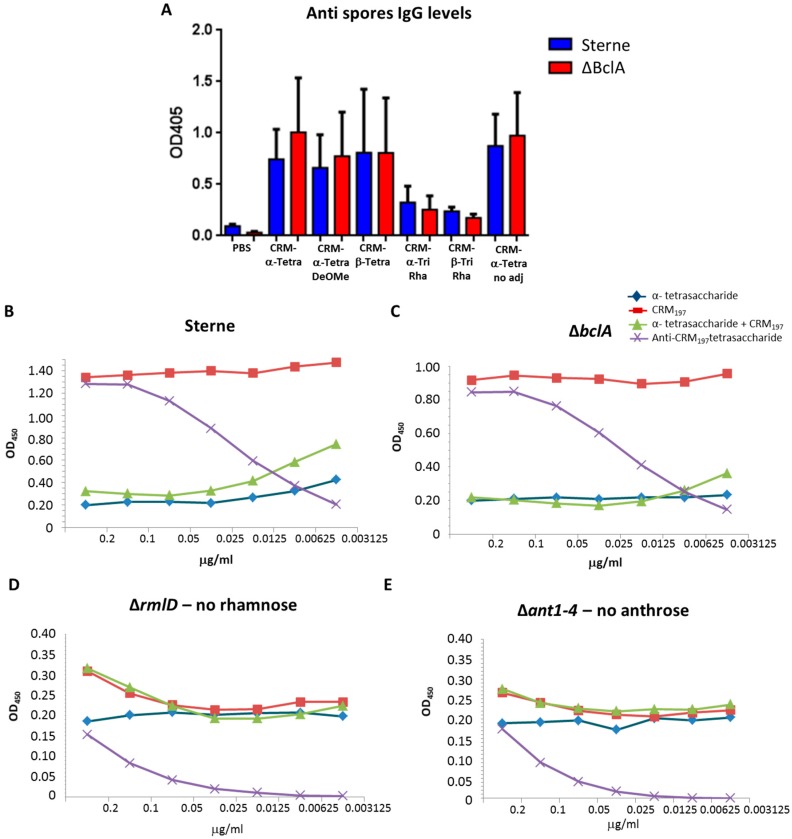
(**A**) Serum Ig anti-*B. anthracis* Sterne spores from mice immunized three times with different conjugated fragments, with and without Alum. (**B**–**E**) Competitive ELISA at different dilutions of α-tetrasaccharide, CRM_197_ and α-tetrasaccharide/CRM_197_ physically mixed with *B. anthracis* spores from Sterne (**B**), Δ*bclA* (**C**), Δ*rmlD* (**D**), or Δ*ant1–4* (**E**).

To assess the capacity of anti-glycan antibodies to recognize the BclA tetrasaccharide expressed on the spore surface, we tested the binding of sera from the mice immunized against spores of *B. anthracis* Sterne, which expresses the full glycoprotein, and *B. anthracis* ΔBclA, a Sterne derivative in which *bclA* was deleted [33] (Figure 6). Sera from mice immunized with anthrose-containing fragments bound to Sterne spores 2–3 fold better than did sera from mice immunized with the tri-rhamnose oligomers (Figure 6A). Unexpectedly, binding of antisera to ΔBclA mutant spores was comparable to binding to Sterne spores. Anti-glycan antisera did not react with spores of the related organism *Bacillus cereus* that does not produce anthrose (Supplemental Information, Figure S1). Taken together, these findings suggest that ΔBclA spores may still produce anthrose linked to another protein, despite absence of the only known anthrose-decorated protein, BclA, on the spore surface.

To determine whether spore recognition was specific, the serum elicited against the conjugated α-tetrasaccharide was pre-incubated with serial dilutions of α-tetrasaccharide, CRM_197_, α-tetrasaccharide + CRM_197_, or anti α-tetrasaccharide + CRM_197_ polyclonal serum. Then the reaction mixtures were applied to microtiter wells coated with spores of *B. anthracis* Sterne, Δ*bclA*, Δ*rmlD*, or Δ*ant1–4* [33]. 

The data showed that free or conjugated tetrasaccharide inhibited binding of anti-tetrasaccharide antibody to the Sterne or Δ*bclA* spores. In contrast, the tetrasaccharide did not affect binding of the anti-glycan antibody to the Δ*rmlD* and Δ*ant1–4* spores. Presumably the anti-tetrasaccharide antibody does not recognize the surface of spores from these two mutant strains because they do not produce rhamnose or anthrose, respectively. Co-incubation of CRM_197_ with the anti-tetrasaccharide antibody did not inhibit binding to the spores, which confirms that the anti-CRM_197_ antibodies that are present in the antisera from mice immunized with the CRM_197_-conjugated glycans do not affect anti-tetrasaccharide binding to the spores.

Globally, these data show that immunization of mice with the two conjugated tetrasaccharide glycoforms in the presence of Alum elicited a robust anti-tetrasaccharide antibody response and that binding of these antibodies to spores that express anthrose can be inhibited by the tetrasaccharide. The conjugated trisaccharides, lacking of the immunodominant anthrose, induced IgG at lower levels compared to the related tetrasaccharides. The adjuvant did not play a relevant role in inducing the immunological response.

## 3. Materials and Methods

### 3.1. Glycoconjugation

Amine derivatives of synthetic saccharides (**1**–**5**, Figure 2) were prepared as reported in the literature [17]. Shortly: saccharides were dissolved in ethylenediamine (2.5 mg/mL solution) and stirred for 48 h under nitrogen. Samples were dried and occurrence of the reaction was assessed by NMR. The compounds were purified by C18 reverse-phase chromatography (water→MeOH). Fractions containing the desired compound, as determined by NMR, were concentrated and used for conjugation to CRM_197_.

The amine-derivatized sugars were dissolved in 9:1 DMSO:H_2_O containing triethylamine (20 equiv). A solution of adipic acid bis(*N*-hydroxysuccinimmide) (SIDEA, 6 equiv) in DMSO was added drop-wise, and the mixture was stirred at RT for 2 h. The activated glycans were purified by precipitation through addition of 20 volumes of ethyl acetate in cold bath (0–4 °C). Suspensions were centrifuged at 4 °C, at 4000× *g* for 5 min and the pellet was washed 7 times with ethylacetate. After quantification of the active NHS groups by colorimetric assay, each fragment was incubated in a 80–100:1 saccharide/protein molar ratio (corresponding to 40–50:1 NHS/protein molar ratio) of a NaPi (Sodium phosphate) 10 mM (pH 7.2) CRM_197_ solution (10 mg/mL) for 16 h at RT. That conjugation had occurred was verified by SDS-page gel electrophoresis (TRIS Acetate, 3–8%) by loading 5 µg of CRM_197_-conjugate for each sample. Conjugates were purified by dialysis (Amicon centrifugal filters, Cut-off 30 kDa) against NaPi buffer (10 mM, pH 7.2). The protein content was determined by standard micro BCA assay.

### 3.2. MALDI-TOF MS Analysis

MALDI-TOF mass spectra were recorded by an UltraFlex III MALDI TOF/TOF instrument (Bruker Daltonics, Dresden, Germany) in linear mode and with positive ion detection. Before analysis, the samples (50 μg) were dialyzed against water via Vivaspin, 30 kDa cut-off. Then 2.5 µL of a product sample was mixed with 2.5 µL of sinapic acid matrix. 2.5 μL of each mixture was applied on the sampling plate, dried at room temperature for 10 min and analyzed.

### 3.3. Immunogenicity of Conjugates in Mice

All animal sera used in this study derived from mouse immunization experiments performed at the Novartis Vaccines Animal Facility in Siena, Italy, (now acquired by the GSK group) in compliance with the relevant guidelines in Italy (Italian Legislative Decree n. 116/1992) and the institutional policies of Novartis (now acquired by the GSK group). The animal protocol was approved by the Animal Welfare Body of Novartis Vaccines, Siena, Italy, (now acquired by the GSK group) and by the Italian Ministry of Health (approval number no. 804/2015-PR). One group of eight female BALB/c mice were immunized by subcutaneous injection of 2 µg dose in saccharide content of each produced CRM_197_ conjugate, using alum hydroxide as an adjuvant. Alum hydroxide in PBS buffer were used as control. Mice received the vaccines at days 1, 14, and 28. Sera were bled at days 0, 27, and 42.

### 3.4. ELISA Analysis Using Bovine Serum Albumin (BSA) Conjugates as Coating Reagent

Microtiter plates (96 wells, NUNC Maxisorp, Thermo Fisher Scientific, Waltham, MA, USA) were coated with 100 μL of 1 μg/mL (dilution in PBS pH 7.4) using two different BSA conjugates as coating reagent: α-tetrasaccharide-BSA or β-tetrasaccharide-BSA. Plates were incubated overnight at 2–8 °C, washed three times with PBST (0.05% Tween-20 in PBS pH 7.4) and saturated with 250 µL/well of PBST-B (2% Bovine Serum Albumin-BSA in PBST) for 90 min at 37 °C. The plates were then aspirated to remove the solution. Two-fold serial dilutions of test and standard sera in PBST-B were added to each well. Plates were then incubated at 37 °C for 2 h, washed with PBST, and then incubated for 90 min at 37 °C with anti-mouse IgG-alkaline phosphatase (Sigma-Aldrich, Saint Louis, MO, USA) diluted 1:2000. After washing, the plates were developed with a 4 mg/mL solution of p-nitrophenyl pPhosphate (pNPP) in 1 M diethanolamine (DEA) pH 9.8, at room temperature for 30 min. After blocking with 7% EDTA, the absorbance was measured using a SPECTRAmax plate reader with wavelength set at 405 nm. IgG titers were calculated as reciprocal serum dilution giving OD of 1.0.

### 3.5. ELISA Analysis to Evaluate Anti-Glycan Binding to B. anthracis Spores

Immulon 2HB “U” bottom microtiter plates (Thermo Fisher Scientific, Waltham, MA, USA) were coated with 10^7^ formalin-inactivated spores of *B. anthracis* strains Sterne (wild-type), Δ*bclA* (gene for BclA, major spore surface protein, deleted), Δ*rmlD* (gene for rhamnose production deleted, no rhamnose produced and no anthrose on surface of spore), Δ*ant1–4* (anthrose biosynthetic genes deleted, no anthrose produced), or *B. cereus* G9241 (no anthrose produced). Plates were coated with spores diluted in PBS overnight at 4 °C. Plates were washed and blocked with 3% BSA in PBS for 2 h at RT. Primary antibodies (anti-glycan) were serially diluted 1:2 in PBS + 0.05% Tween (PBST), applied to the plates, and incubated 1 h at RT. Following 4 washes with PBST, goat anti-mouse IgG (Bio-Rad, Hercules, CA, USA) was diluted 10^−4^ in PBS, applied to the plates, and incubated for 1 h at RT. Plates were again washed 4 times with PBST and TMB peroxidase EIA substrate was applied. The reactions were stopped with an equal volume of 1 N sulfuric acid and the absorbance of each well was read at 450 nm.

For competitive inhibition, ELISA plates were coated and blocked as above. During the blocking step, the inhibition reactions were carried out. Sera from mice immunized with conjugated α-tetrasaccharide were diluted 1:40 and mixed with serial two-fold dilutions of α-tetrasaccharide (2 mg/mL starting), CRM_197_ (0.2 mg/mL starting), both α-tetrasaccharide and CRM_197_ (same starting concentrations), or PBS. These reactions were incubated at 37 °C for 2 h, then applied to the blocked plates. The rest of the procedure was as above.

## 4. Conclusions

The tetrasaccharide from the major exosporium protein (BclA) of *B. anthracis* has been proposed as a target for development of diagnostics, therapeutics, or vaccines. The use of a synthetic carbohydrate appears particularly attractive in this case to obviate large scale extraction of carbohydrates from spore forming bacteria. In addition, gram-scale production of this tetrasaccharide has been shown feasible [24].

While the role of the upstream anthrose residue in the immunogenicity of the tetrasaccharide from the most abundant *B. anthracis* exosporium protein has been thoroughly explored, little is known on the configuration of the linkage to the GlcNAc residue bridging the glycan and the protein.

By immuniziation of mice with the prepared glycoconjugates we showed that the anti α-tetrasaccharide serum recognized the antigen which elicited it in a similar manner as its β-glycoform, and vice versa. In contrast, glycoconjugates made from each of the two α- and β-trirhamnoside fragments elicited sera which were more specific for antigens that had given rise to them than to the serum produced by its anomeric counterpart. This result confirmed the immunodominant character of anthrose, which tends to drive the immune response towards this residue. 

Importantly, the anti α- and β-tetrasaccharide sera also recognized Sterne spores in a comparable fashion. Surprisingly, ΔBclA spores not expressing the major exosporium protein were also recognized, while mutants with deletion of either rhamnose or anthrose were not. The tetrasaccharide could, therefore, be expressed in proteins other than BclA, and this aspect would deserve further exploration.

Our results indicate that both α- and β-glycoform of the tetrasaccharide could be used as antigen for immunoprophylaxis or immunotherapy against *B. anthracis.*

## 5. Patents

R.S., P.K., and R.A. are owners of a patent related to this topic.

## Supplementary Materials

The Figure S1 is available online.

## References

[1] Hanna P. (1998). Anthrax pathogenesis and host response. Curr. Top. Microbiol. Immunol..

[2] Wang J.Y., Roehrl M.H. (2005). Anthrax vaccine design: Strategies to achieve comprehensive protection against spore, bacillus, and toxin. Med. Immunol..

[3] Webb G. (2005). Being prepared: Modeling the response to an anthrax attack. Ann. Intern. Med..

[4] Young J.A., Collier R.J. (2007). Anthrax toxin: Receptor binding, internalization, pore formation, and translocation. Annu. Rev. Biochem..

[5] Bouzanias D.G. (2009). Medical countermeasures to protect humans from anthrax bioterrorism. Trends Microbiol..

[6] Daubenspeck J.M., Zeng H., Chen P., Dong S., Steichen C.T., Krishna N.R., Pritchard D.G., Turnbough C.L. (2004). Novel oligosaccharide side chains of the collagen-like region of bcla, the major glycoprotein of the bacillus anthracis exosporium. J. Biol. Chem..

[7] Berti F., Adamo R. (2013). Recent mechanistic insights on glycoconjugate vaccines and future perspectives. ACS Chem. Biol..

[8] Astronomo R.D., Burton D.R. (2010). Carbohydrate vaccines: Developing sweet solutions to sticky situations?. Nat. Rev. Drug Discov..

[9] Adamo R. (2014). Glycan surface antigens from Bacillus anthracis as vaccine targets: Current status and future perspectives. Exp. Rev. Vaccines.

[10] Saksena R., Adamo R., Kováč P. (2005). Studies toward a conjugate vaccine for anthrax. Synthesis and characterization of anthrose [4,6-dideoxy-4-(3-hydroxy-3-methylbutanamido)-2-o-methyl-d-glucopyranose] and its methyl glycosides. Carbohydr. Res..

[11] Milhomme O., Grandjean C. (2014). Synthetic efforts towards glycoconjugate-based vaccines active against anthrax. Curr. Org. Synth..

[12] Oberli M.A., Horlacher T., Werz D.B., Seeberger P.H., Kosma P., Müller-Loennies S. (2012). Synthetic oligosaccharide bacterial antigens to produce monoclonal antibodies for diagnosis and treatment of disease using bacillus anthracis as a case study. Anticarbohydrate Antibodies.

[13] Werz D.B., Seeberger P.H. (2005). Total synthesis of antigen bacillus anthracis tetrasaccharide—Creation of an anthrax vaccine candidate. Angew. Chem. Int. Ed. Engl..

[14] Adamo R., Saksena R., Kováč P. (2005). Synthesis of the beta anomer of the spacer-equipped tetrasaccharide side chain of the major glycoprotein of the bacillus anthracis exosporium. Carbohydr. Res..

[15] Saksena R., Adamo R., Kováč P. (2006). Synthesis of the tetrasaccharide side chain of the major glycoprotein of the bacillus anthracis exosporium. Bioorg. Med. Chem. Lett..

[16] Adamo R., Saksena R., Kováč P. (2006). Studies towards a conjugate vaccine for anthrax: Synthesis of the tetrasaccharide side chain of the bacillus anthracis exosporium. Helv. Chim. Acta.

[17] Saksena R., Adamo R., Kováč P. (2007). Immunogens related to the synthetic tetrasaccharide side chain of the bacillus anthracis exosporium. Bioorg. Med. Chem..

[18] Mehta A.S., Saile E., Zhong W., Buskas T., Carlson R., Kannenberg E., Reed Y., Quinn C.P., Boons G.J. (2006). Synthesis and antigenic analysis of the Bcla glycoprotein oligosaccharide from the *Bacillus anthracis* exosporium. Chemistry.

[19] Crich D., Vinogradova O. (2007). Synthesis of the antigenic tetrasaccharide side chain from the major glycoprotein of *Bacillus anthracis* exosporium. J. Org. Chem..

[20] Guo H., O’Doherty G.A. (2007). De novo asymmetric synthesis of the anthrax tetrasaccharide by a palladium-catalyzed glycosylation reaction. Angew. Chem. Int. Ed. Engl..

[21] Dhenin S.G., Moreau V., Morel N., Nevers M.C., Volland H., Creminon C., Djedaini-Pilard F. (2008). Synthesis of an anthrose derivative and production of polyclonal antibodies for the detection of anthrax spores. Carbohydr. Res..

[22] Hou S., Kováč P. (2008). A convenient synthesis of furanose-free d-fucose per-o-acetates and a precursor for anthrose. Eur. J. Org. Chem..

[23] Milhomme O., Dhenin S.G., Djedaini-Pilard F., Moreau V., Grandjean C. (2012). Synthetic studies toward the anthrax tetrasaccharide: Alternative synthesis of this antigen. Carbohydr. Res..

[24] Hou S., Kováč P. (2009). Conjugation-amenable tetrasaccharide of the side chain of the major glycoprotein of the bacillus anthracis exosporium: A large-scale preparation. Synthesis.

[25] Werz D.B., Adibekian A., Seeberger P.H. (2007). Synthesis of a spore surface pentasaccharide of bacillus anthracis. Eur. J. Org. Chem..

[26] Tamborrini M., Werz D.B., Frey J., Pluschke G., Seeberger P.H. (2006). Anticarbohydrate antibodies for the detection of anthrax spores. Angew. Chem. Int. Ed. Engl..

[27] Wang D., Carroll G.T., Turro N.J., Koberstein J.T., Kovac P., Saksena R., Adamo R., Herzenberg L.A., Steinman L. (2007). Photogenerated glycan arrays identify immunogenic sugar moieties of bacillus anthracis exosporium. Proteomics.

[28] Oberli M.A., Tamborrini M., Tsai Y.-H., Werz D.B., Horlacher T., Adibekian A., Gauss D., Moller H.M., Pluschke G., Seeberger P.H. (2010). Molecular analysis of carbohydrate-antibody interactions: Case study using a bacillus anthracis tetrasaccharide. J. Am. Chem. Soc..

[29] Milhomme O., Köhler S.M., Ropartz D., Lesur D., Pilard S., Djedaïni-Pilard F., Beyerb W., Grandjean C. (2012). Synthesis and immunochemical evaluation of a non-methylated disaccharide analogue of the anthrax tetrasaccharide. Org. Biomol. Chem..

[30] Micoli F., Adamo R., Costantino P. (2018). Protein carriers for glycoconjugate vaccines: History, selection criteria, characterization and new trends. Molecules.

[31] Adamo R., Romano M.R., Berti F., Leuzzi R., Tontini M., Danieli E., Cappelletti E., Cakici O.S., Swennen E., Pinto V. (2012). Phosphorylation of the synthetic hexasaccharide repeating unit is essential for the induction of antibodies to Clostridium difficile PSII cell wall polysaccharide. ACS Chem. Biol..

[32] Xu P., Trinh M.N., Kováč P. (2018). Conjugation of carbohydrates to proteins using di (triethylene glycol monomethyl ether) squaric acid ester revisited. Carbohydr. Res..

[33] Swiecki M.K., Lisanby M.W., Shu F., Turnbough C.L., Kearney J.F. (2006). Monoclonal antibodies for bacillus anthracis spore detection and functional analyses of spore germination and outgrowth. J. Immunol..

