# Supplementary material for: Structure-Immunogenicity Relationship of α- and β-Tetrasaccharide Glycoforms from Bacillus anthracis Exosporium and Fragments Thereof
Source: Molecules. 2018 Aug 20;23(8):2079. doi: 10.3390/molecules23082079 (PMC6222408; doi:10.3390/molecules23082079)
Supplement: Supplementary file 1 [file molecules-23-02079-s001.pdf]

Supplemental information

Structure-immunogenicity relationship of α- and β-tetrasaccharide glycoforms from *Bacillus anthracis* exosporium and fragments thereof

Riccardo De Ricco [1], Christy L. Ventura[2], Filippo Carboni [1], Rina Saksena [3,4], Pavol Kováč [3], Roberto Adamo [1*]

[1] GSK, Research Centre, via Fiorentina 1, 53100 Siena, Italy; riccardo.x.de-ricco@gsk.com; filippo.x.carboni@gsk.com

[2] Department of Microbiology and Immunology, Uniformed Services University of the Health Sciences, Bethesda, MD 20814; christy.ventura.ctr@usuhs.edu

[3] NIDDK, LBC, National Institutes of Health, Bethesda, MD 20892-0815; kovac@niddk.nih.gov

[4] Current Address: The University of New Mexico, Department of Chemistry, Albuquerque, NM 87131; rsaksena@unm.edu.

* Correspondence: roberto.x.adamo@gsk.com; Tel.: +39 0577 539393

Anti Bacillus cereus antibody levels

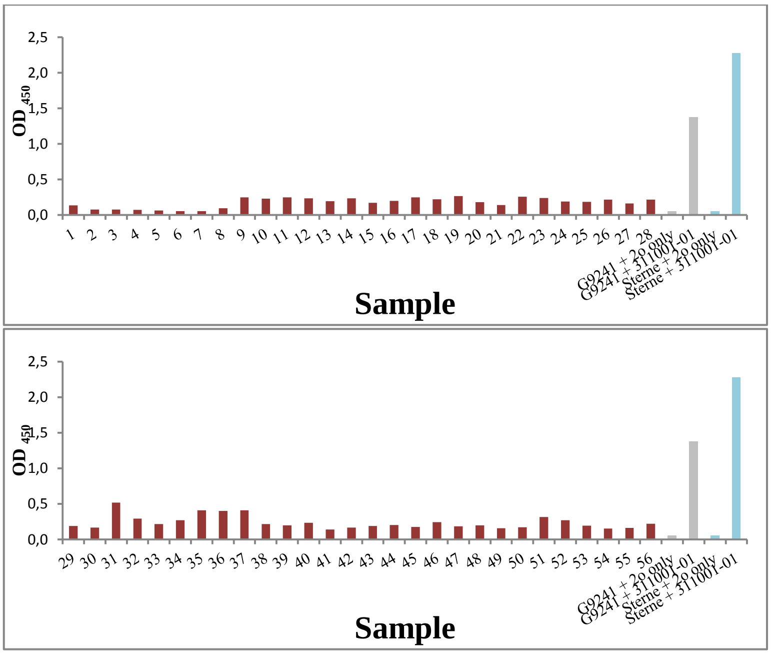

Figure S1. Reactivity of anti conjugate sera with *B. cereus* G9241 spores of sera diluted 1:160.
